# Jaceidin Suppresses Melanoma Metastasis by Modulating ERK, JNK Pathways and Extracellular Matrix Degradation Proteins

**DOI:** 10.1111/jcmm.71068

**Published:** 2026-02-23

**Authors:** Mu‐Kuei Shieu, Hui‐Ju Yang, Chia‐Chieh Lin, Min‐Yun Kao, Hsin‐Yu Ho, Yu‐Sheng Lo, Yi‐Ching Chuang, Yuan‐Ting Yang, Ming‐Ju Hsieh

**Affiliations:** ^1^ Department of Dermatology Changhua Christian Hospital Changhua Taiwan; ^2^ Oral Cancer Research Center Changhua Christian Hospital Changhua Taiwan; ^3^ Department of Pharmacy Changhua Christian Hospital Changhua Taiwan; ^4^ Graduate Institute of Clinical Medicine, College of Medicine National Chung Hsing University Taichung Taiwan; ^5^ Doctoral Program in Tissue Engineering and Regenerative Medicine, College of Medicine National Chung Hsing University Taichung Taiwan; ^6^ Graduate Institute of Biomedical Sciences China Medical University Taichung Taiwan

**Keywords:** cathepsins, invasion, jaceidin, melanoma, migration, MMP‐2

## Abstract

Metastatic melanoma, marked by its poor prognosis and frequent recurrence, is a particularly aggressive skin cancer. Jaceidin, a compound derived from flavonoids abundant in common fruits and vegetables, has attracted interest for its potential anti‐cancer properties. Nevertheless, the specific effects of jaceidin on melanoma cells remained unclear before this study. Here, we examined the impact of jaceidin on two metastatic melanoma cell lines, HMY‐1 and A2058. Our results demonstrate that jaceidin exerts a significant anti‐metastatic effect against both cell lines. This inhibitory action involved modulating the phosphorylation of extracellular signal‐regulated kinase and c‐Jun N‐terminal kinase, as well as suppressing proteins associated with epithelial‐mesenchymal transition. Furthermore, jaceidin reduced the expression of extracellular matrix degradation proteins MMP‐2 and cathepsins A. These compelling findings suggest that jaceidin warrants further investigation as a potential therapeutic agent for melanoma.

## 1 Introduction

Among skin cancer, cutaneous melanoma has remained the most significant global health concerns for decades. In 2024, it is estimated that 100,000 new cases will be diagnosed in the United States alone [1]. Additionally, the annual incidence of melanoma has been steadily increasing in most countries, according to the World Health Organization (WHO) database [2]. Various risk factors have been identified in the development of melanoma, including over‐exposure to ultraviolet, family history of the disease, genetic predisposition and ethnicity, with white individuals being particularly at risk [3, 4]. Furthermore, melanoma can be diagnosed at any age, with cases reported in both neonates and the elderly [5].

While most of the melanoma tumour are classified as primary stages (I and II) [6], they can typically be treated effectively through wide local excision [7], with survival rate extending to 10 years or longer [8]. In contrast, once advanced stage of melanoma (III and IV) was diagnosed, it becomes a significant challenge for both clinicians and patients due to its highly metastatic rate and recurrence. According to previous studies, before the systematic treatment was invented, the median survival of advanced melanoma only lasted for 6 months [9]. Fortunately, recent years have seen the emergence of several new treatment options, including the application of immunotherapy (e.g., Cytotoxic T‐lymphocyte‐associated antigen‐4, Programmed Cell Death Protein 1, or Programmed Cell Death Ligand 1), target therapy (e.g., BRAF inhibitors) or chemotherapy. While these therapies have led to significant improvements in survival rates [10], the adverse effects, including high toxicity, remain a considerable concern [11, 12]. Furthermore, the following financial cost during therapeutic course has become a burden for both patients and their families [13].

Fortunately, increasing research efforts are being directed towards identifying new therapeutic pathways to combat melanoma. One such focus is the epithelial–mesenchymal transition (EMT), which plays a critical role in melanoma progression and is closely associated with enhanced cell motility and invasiveness [14]. For instance, one study demonstrated that green tea polyphenols can inhibit the proliferation of A875 and SK‐MEL‐1 melanoma cells by downregulating EMT‐related gene expression [15, 16], Another study found that long pentraxin‐3, a component of the fibroblast growth factor family, suppresses the proliferation of A375 and A2058 melanoma cells through similar downregulation of EMT markers [17].

In the present study, we focused on an organic compound called jaceidin as our main target against melanoma cells. Jaceidin is a member of the flavonoid family, which is commonly found in fruits and vegetables [18]. Flavonoids can be categorized into several subclasses [19], with some derivatives documented to exhibit anti‐cancer properties, particularly in inhibiting invasion and migration in various cancer cells [20]. For example, in gastric cancer cells, a compound called jaceosidin was shown to suppress cell migration by activating reactive oxygen species‐mediated signalling pathways [21]. In cervical cancer cells, a synthetic compound, WYC02‐9, was found to promote apoptosis and inhibit cell migration by activating the mitogen‐activated protein kinase 14 pathway [22]. One previous study highlighted jaceidin's ability to suppress ascites carcinoma progression in mice, resulting in a significant reduction in tumour weight [23]. Furthermore, jaceidin and other flavonoid derivatives have been shown to exert anti‐carcinogenic properties by inducing cell apoptosis in breast cancer [24].

To date, however, the effects of jaceidin on melanoma cells remain unexplored. Therefore, this study aims to investigate the potential chemotherapeutic effects of jaceidin on the invasion and migration of malignant melanoma cells.

## 2 Material and Methods

### 2.1 Cell Culture

Two melanoma cell lines, HMY‐1 (BRAF wild type) and A2058 (BRAF mutant), were selected as the target cells for this study. These cell lines were derived from metastatic lymph nodes of a 62‐year‐old male and a 43‐year‐old male [25, 26]. Both lines were obtained from the Japanese Collection of Research Bioresources (Tokyo, Japan). The cells were cultured in Dulbecco's Modified Eagle Medium (Gibco BRL, Grand Island, NY, USA), supplemented with 10% foetal bovine serum and 1% penicillin/streptomycin. The cells were then incubated at 37°C in a 5% CO_2_ atmosphere under humidified conditions.

### 2.2 Chemical Treatments

Jaceidin (C18H16O8, molecular weight: 360.31, ≥ 96% purity) was purchased from ChemFaces (Wuhan, China). A stock solution of jaceidin (100 μM) was prepared using dimethyl sulfoxide (DMSO) and stored at −20°C for future use. Based on the required treatment doses, the stock solution was serially diluted to prepare working solutions. The final concentration of DMSO in all treatments was maintained at less than 0.1%.

### 2.3 MTT Assay

To assess cell viability, melanoma cells were cultured in 24‐well plates and treated with 25, 50, or 100 μM jaceidin for 24 h. Untreated cells served as the control group. After incubation, the MTT assay (3‐(4,5‐dimethylthiazol‐2‐yl)‐2,5‐diphenyltetrazolium bromide) was performed to evaluate cell viability. DMSO was then added to dissolve the purple crystals, and absorbance was measured using a BioTek spectrophotometer (Winooski, VT, USA) at 570 nm.

### 2.4 Wound Closure Assay

Two melanoma cell lines (HMY‐1 and A2058) were seeded into 12‐well culture plates and incubated overnight to reach 90% confluence. After creating a wound using a tip, the cells were incubated with different concentrations of jaceidin (25, 50, 100 μM) for 3, 6 and 24 h. Wound photographs and the crawling distance of cells were recorded under a microscope.

### 2.5 Cell Migration and Invasion Assay

To analyse cell migration and invasion, transwell assays were performed using transwell inserts. The cells were treated with different concentrations of jaceidin (25, 50 and 100 μM) for 24 h. After 24 h of incubation, the cells were fixed with methanol for 10 min, then dried. The cells were stained with 10× Giemsa for 4 h, and the non‐migrating cells on the upper surface of the membrane were removed. The number of migrating cells was then counted under a 100X microscope. For the invasion assay, the procedure was repeated with a 10‐fold dilution, and the steps were the same as the migration assay, and diluted Matrigel gel (25 mg/50 mL; 60 μL; BD Biosciences) was coated on the upper transwell at 37°C for overnight.

### 2.6 Western Blot Assay

For Western blot analysis, protein samples were extracted using lysis buffer. The samples were then separated by 10% polyacrylamide gel electrophoresis and transferred to polyvinylidene fluoride (PVDF) membranes (Millipore Corporation, Milford, MA). The membranes were blocked with milk for 1 h, followed by incubation with primary antibodies for 24 h. Afterward, the membranes were incubated with secondary antibodies at room temperature for 1 h. Finally, protein bands were visualized using the ImageQuant fluorescence biomolecule imaging system.

### 2.7 Statistical Analysis

Statistical analysis was conducted using SigmaPlot v12.5 (Systat Software; Palo Alto, CA, USA). A *p* < 0.05 was considered statistically significant. One‐way analysis of variance (ANOVA) followed by Tukey's multiple comparison test was used to compare the treated cells with the control group.

## 3 Results

### 3.1 Effect of Jaceidin on Cell Viability in Melanoma Cell Lines

**FIGURE 1:**
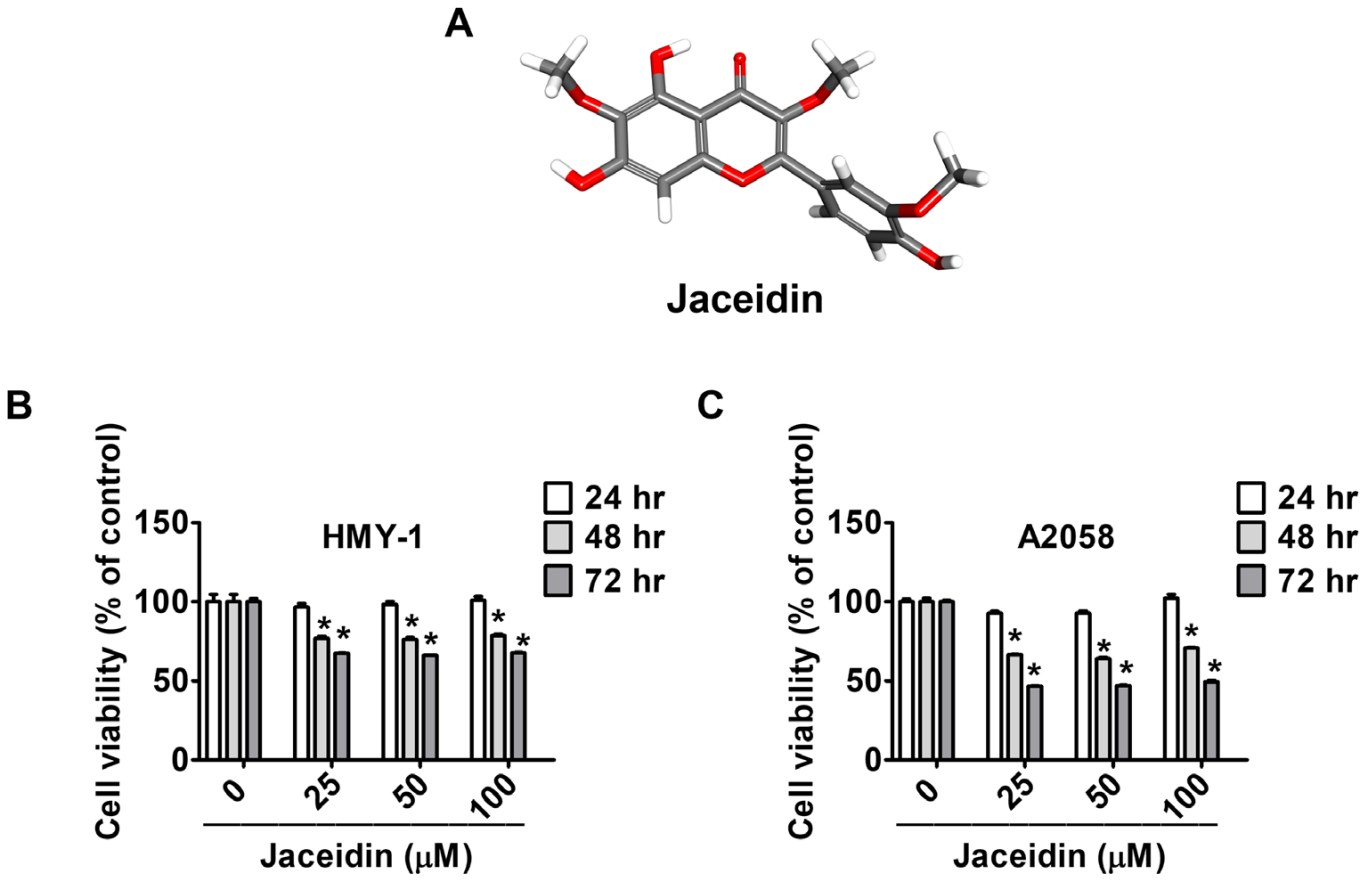
Toxicity of jaceidin against melanoma cells. (A) Chemical structure of jaceidin. (B, C) MTT assay of HMY‐1 and A2058 cells treated with different concentrations of jaceidin (0, 25, 50 and 100 μM) for 24, 48 and 72 h. Data are mean ± SD of three independent experiments. *p < 0.05, compared with vehicle.

Figure 1A shows the chemical structure of jaceidin. To investigate the cytotoxicity of jaceidin, the HMY‐1 and A2058 melanoma cell lines were treated with different concentrations (25, 50 and 100 μM) of jaceidin for 24, 48 and 72 h. MTT assays were performed as shown in Figure 1B,C. DMSO treated cells served as the control group. The results indicated that jaceidin concentrations of 25, 50 and 100 μM did not exhibit cytotoxicity after 24 h of treatment. However, significant cytotoxicity was observed after 48 and 72 h of treatment, in comparison to the DMSO treated group. These findings suggest that jaceidin effectively inhibits the viability of melanoma cells, with prolonged exposure leading to enhanced cytotoxic effects.

### 3.2 Effect of Jaceidin on Cell Motility in Melanoma Cell Lines

**FIGURE 2:**
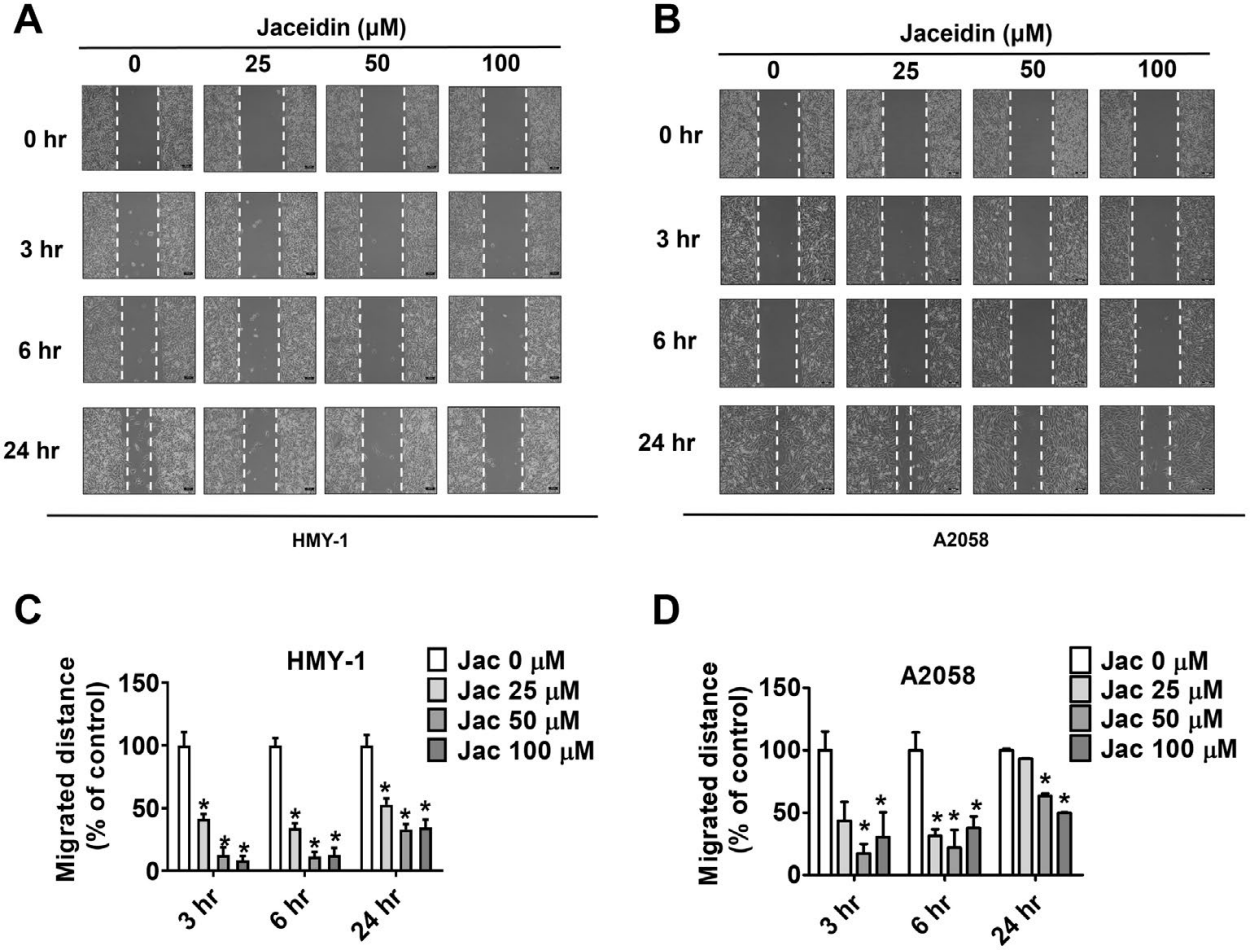
Jaceidin inhibited horizontal migration of melanoma cells. (A, C) HMY‐1 cells treated with indicated concentrations of jaceidin (0, 25, 50 and 100 μM) and photographed at 0, 3, 6 and 24 h to measure distance migrated. (B, D) A2058 cells treated with indicated concentrations of jaceidin (0, 25, 50 and 100 μM) and photographed at 0, 3, 6 and 24 h to measure distance migrated. Data are mean ± SD of three independent experiments. *p < 0.05, compared with vehicle.

To evaluate cell motility, a wound healing assay was performed. As described earlier, HMY‐1 and A2058 cells were treated with 25, 50 and 100 μM of jaceidin for 3, 6 and 24 h, respectively. As shown in Figure 2, jaceidin reduced the migration distance of both melanoma cell lines in a dose‐dependent manner.

### 3.3 Effect of Jaceidin on Invasion and Migration in Melanoma Cell Lines

**FIGURE 3:**
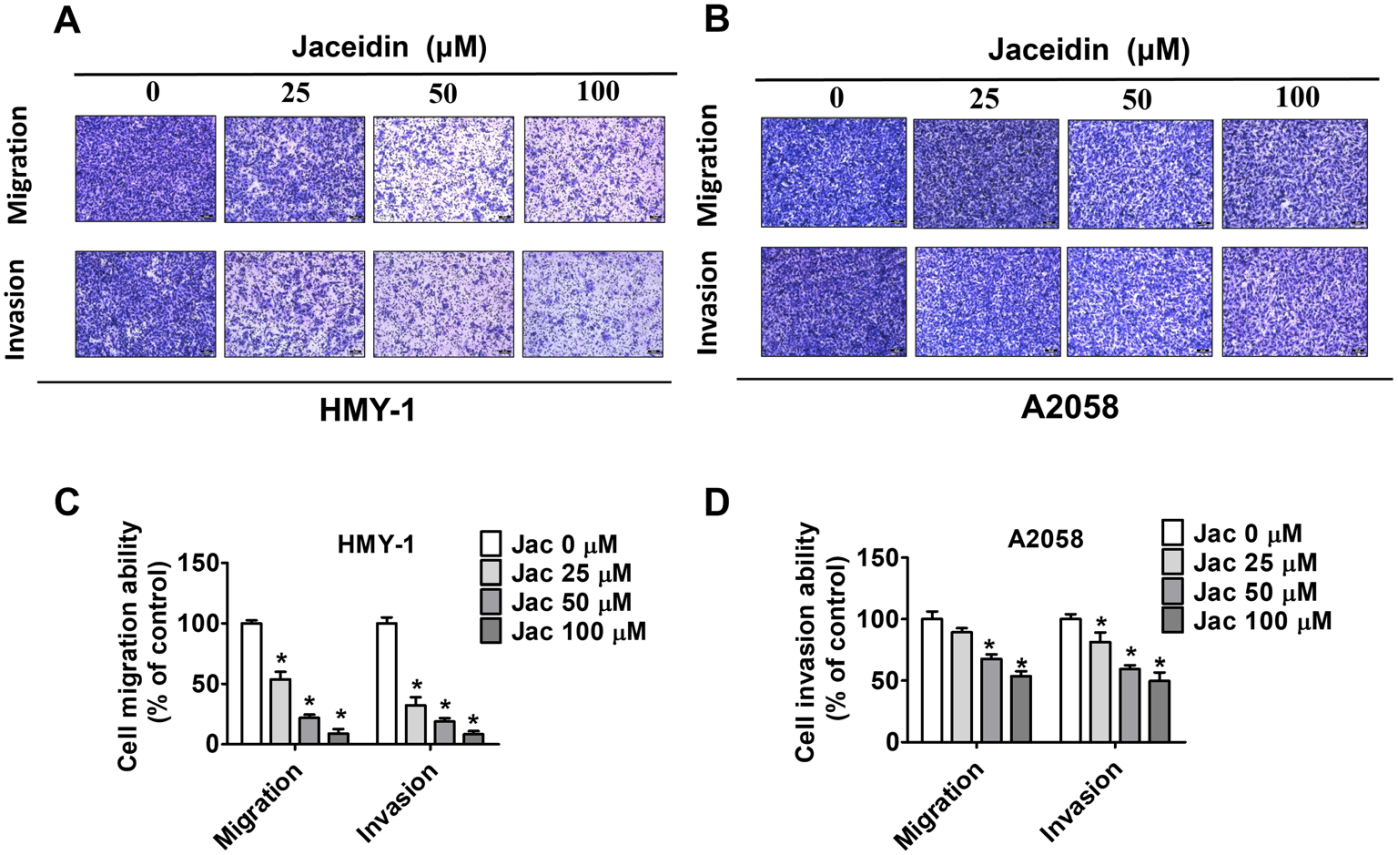
Jaceidin inhibited migration and invasion through melanoma cells (A, C) migration and invasion assays for HMY‐1 cells (B, D) migration and invasion assays for A2058 cells. Cells stained with Giemsa staining buffer were counted to analyse cell migration and invasion. Data are mean ± SD of three independent experiments. *p < 0.05, compared with vehicle.

In our study, the effects of jaceidin on cell invasion and migration were assessed using the transwell assay. As shown in Figure 3, jaceidin treatment significantly inhibited both invasion and migration in both melanoma cell lines, with a particularly notable reduction observed in HMY‐1 cells. Overall, these findings suggest that jaceidin exerts anti‐metastatic effects on malignant melanoma cell lines.

### 3.4 Jaceidin Reduces Protein Expression in the MAPK Pathways of Melanoma Cell Lines

**FIGURE 4:**
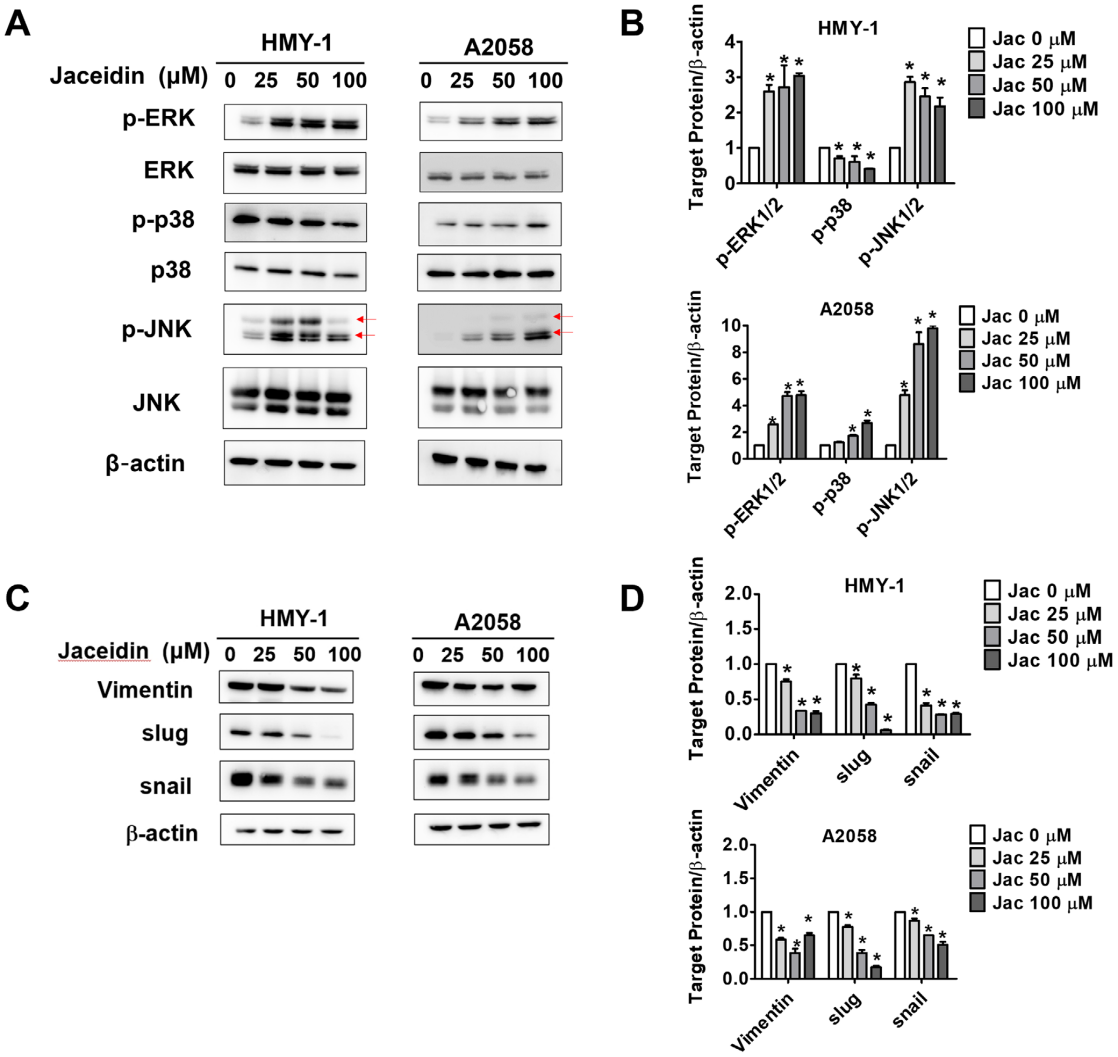
MAPK pathways involved in jaceidin‐induced metastasis regulation in melanoma cells. (A, B) After jaceidin treatment, total and phosphorylated ERK, p38 and JNK levels in HMY‐1 and A2058 cells were measured using Western blot assay with β‐Actin as an internal control. (C, D) Expressions of EMT‐related proteins (vimentin, slug and snail) were measured using Western blot assay with *β*‐Actin as an internal control. Data are mean ± SD of three independent experiments. *p < 0.05, compared with vehicle; #*p* < 0.05, compared with jaceidin only.

The MAPK pathway is well‐known for its strong association with cell proliferation and migration [27]. To explore the signalling pathways involved in the effects of jaceidin on cell migration, three key MAPK pathways were selected as targets: extracellular signal‐regulated kinase (ERK), p38 and c‐Jun N‐terminal kinase (JNK) [19]. In this study, as the concentration of jaceidin increased, the phosphorylation of ERK and JNK pathways significantly increased in both melanoma cell lines. In contrast, the phosphorylation of the p38 pathway decreased in HMY‐1 cells, with no statistically significant change observed in the A2058 cell line (Figure 4A,B). As previously mentioned, EMT plays a crucial role in enhancing cell mobility and invasion [28, 29]. Therefore, we examined the expressions of EMT‐related proteins, including vimentin [30], slug [31] and snail [32]. Our results showed that jaceidin reduced the expression of these proteins (Figure 4C,D). These findings suggest that jaceidin inhibits melanoma cell metastasis by modulating the phosphorylation of ERK, JNK, as well as downregulating EMT‐related proteins.

### 3.5 Effects of Jaceidin co‐Treatment With U0126 and JNK‐IN‐8 on Melanoma Cell Lines

**FIGURE 5:**
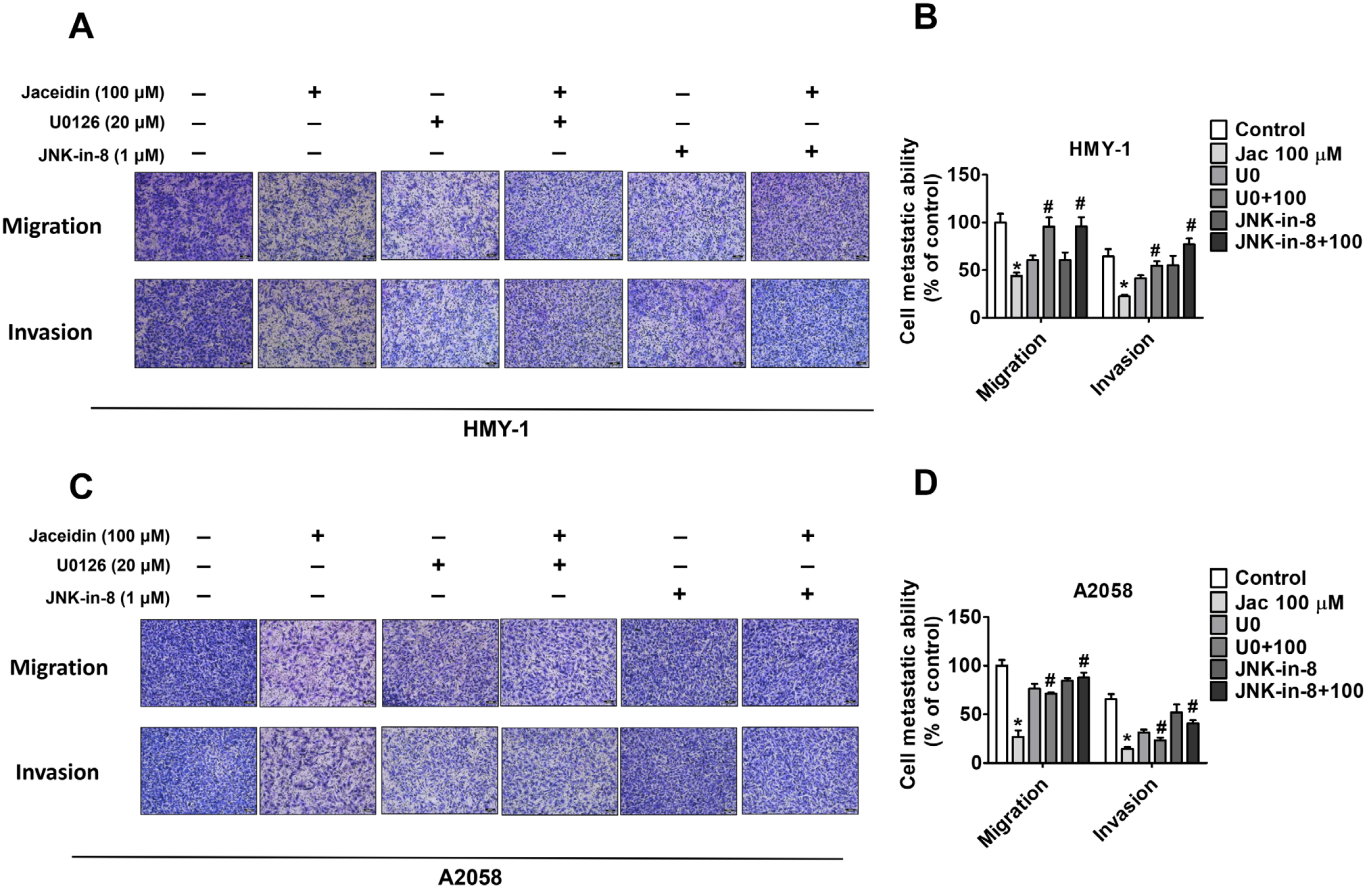
ERK and JNK pathway participated in jaceidin‐induced metastasis regulation. (A, B) HMY‐1 and (C, D) A2058 cells treated with ERK inhibitor (U0126) and JNK inhibitor (JNK‐IN‐8), respectively, for 1 h. Cells were cotreated (or not) with 100 μM jaceidin for 24 h and subjected to migration and invasion assays. Data are mean ± SD of three independent experiments. *p < 0.05, compared with vehicle; #*p* < 0.05, compared with jaceidin only.

To further investigate the involvement of the ERK and JNK pathways in cell migration and invasion, small molecule inhibitors—U0126 (an ERK inhibitor) and JNK‐IN‐8 (a JNK inhibitor)—were used. As shown in Figure 5, the results demonstrated that, in HMY‐1 cells, co‐treatment with jaceidin, U0126 and JNK‐IN‐8 significantly enhanced migration and invasion compared to the control groups. Similarly, in A2058 cells, the co‐treatment produced comparable results. Taken together, these findings suggest that jaceidin inhibits melanoma cell metastasis by upregulating both the ERK and JNK pathways.

### 3.6 Effects of Jaceidin on MMP and Cathepsin A Expression in Melanoma Cells

**FIGURE 6:**
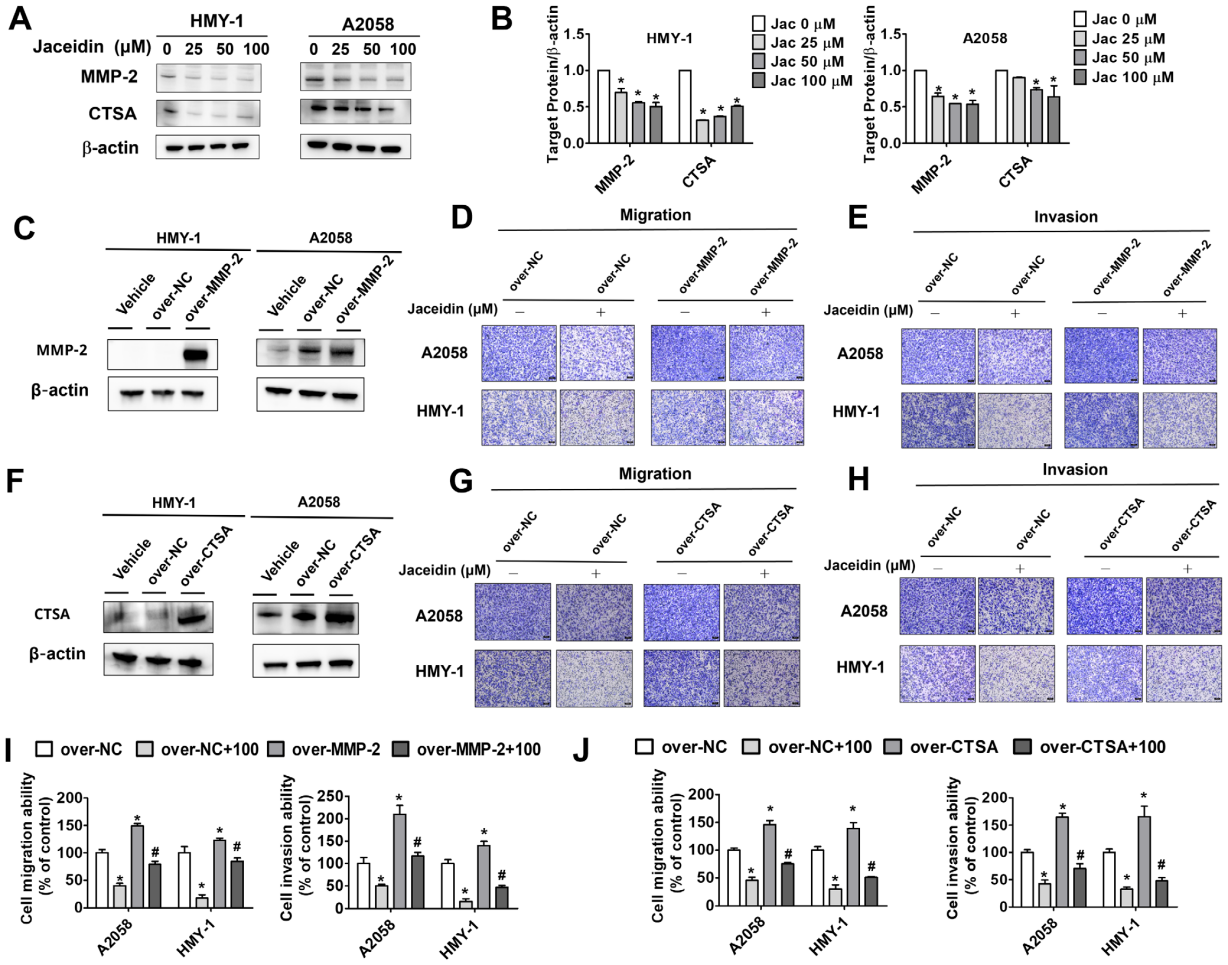
Jaceidin regulated cell metastasis by downregulating MMP‐2 and CTSA in melanoma cells. (A, B) MMP‐2 and CTSA were measured using Western blot assay after jaceidin treatment (0, 25, 50, or 100 μM), with β‐Actin as internal control. (C, D, E, I) Cells were transfected with pEGFP (empty vector) or MMP‐2 overexpression plasmid following treatment with or without jaceidin (100 μM), with β‐Actin as internal control. (F, G, H, J) Cells were transfected with pEGFP (empty vector) or CTSA overexpression plasmid following treatment with or without jaceidin (100 μM). Migration and invasion assays were used to analyse HMY‐1 and A2058 cells. *p < 0.05, compared with vehicle; #*p* < 0.05, compared with jaceidin only.

The degradation of the extracellular matrix (ECM) is a critical step in tumour metastasis [33], with both matrix metalloproteinases (MMPs) [34] and cathepsin family proteins [35] playing vital roles in this process. Among the MMP family, MMP‐2 and MMP‐9 are widely recognized as cancer biomarkers [36]. As shown in Figure 6A,B, treatment with varying concentrations of jaceidin significantly inhibited the expression of MMP‐2 and CTSA in both melanoma cell lines. The down‐regulation of MMP‐2 and CTSA indicates that both have vital roles in the antimetastatic effects of jaceidin. In Figure 6C,F, MMP‐2 and CTSA levels were up‐regulated through transfection of the MMP and CTSA plasmids. In addition, cells cotreated with over‐expression of MMP‐2 and CTSA, respectively, exhibited higher motility compared with the control group (Figure 6D,H), and the motility was inhibited after treatment with jaceidin (Figure 6I,J). Overall, the results indicated that CTSA and MMP‐2 took part in the antimetastatic properties of jaceidin in both melanoma cell lines.

## 4 Discussion

Natural products have been used as therapeutic options since ancient times [37], often under the umbrella of Traditional Chinese Medicine (TCM) or folk medicine in Asia, with many having been documented for thousands of years. Even today, numerous natural compounds continue to be explored for their medicinal potential [38]. These phytochemicals have shown distinct anti‐tumour effects against various cancers [39, 40]. In this study, we found that jaceidin, a flavonoid compound, effectively inhibited the growth of two metastatic melanoma cell lines, HMY‐1 and A2058. Our results showed that jaceidin significantly reduced melanoma cell viability. Furthermore, wound healing and transwell assays indicated that jaceidin's effects were associated with the upregulation of the ERK and JNK pathways, along with reduced expression of EMT and MMP‐related proteins. While few studies have explored the synergistic effects of jaceidin, as mentioned previously [23]. To the best of our knowledge, this is the first study to demonstrate that jaceidin inhibits cell migration and invasion in melanoma cell lines.

Among the initial steps of tumour progression, EMT is a dynamic process in which epithelial cells lose their epithelial characteristics and transform into mesenchymal cells [41]. Mesenchymal cells contribute to the promotion of tumour growth [14, 42]. This phenomenon can occur in both epithelial and non‐epithelial cancers, including melanoma [43]. As members of the transcriptional factor family, slug and snail have been shown to induce EMT in various cancer cells [44]. In ovarian cancer cells, they have been associated with increased radio resistance and chemo resistance to paclitaxel and cisplatin [45]. In B16 melanoma cells, studies have found that snail and slug are key regulators of nodal‐induced EMT in mice [46], where overexpression of nodal enhances tumour migration. Our study suggests that jaceidin is involved in downregulating the expression of EMT‐related proteins.

Considering the upstream signalling pathways in the regulation of cells, the MAPK pathway plays a crucial role in regulating various cellular processes, including cell proliferation, migration and apoptosis in mammalian cells [27, 47, 48]. We discovered that jaceidin inhibits the invasion and migration of A2058 and HMY‐1 melanoma cells by upregulating both the ERK and JNK signalling pathways. Interestingly, current BRAF inhibitors, such as vemurafenib, dabrafenib and encorafenib [49], are being used in combination with immunotherapy to target advanced stages of melanoma [50]. These inhibitors primarily target the ERK pathway, effectively inhibiting cell proliferation and improving survival outcome [51]. However, resistance mechanisms against BRAF inhibitors have been described, with reactivation of the MAPK pathway noted after approximately 12 months of treatment [52, 53]. This resistance has been specifically observed in BRAF‐mutant A2058 cell lines [54, 55]. Although our study highlights contrasting mechanisms by inhibiting melanoma cell migration through activation of the ERK and JNK pathways, other natural products have also yielded similar results. In B16F10 melanoma cells, theaflavin inhibited migration and invasion via the upregulation of JNK, p53 and downregulation of the ERK pathway [56]. Another natural compound, Gomisin A, also inhibited migration and invasion through activation of the MAPK, ERK and JNK pathways in B16F10 and A375 cell lines [57]. Conversely, Taxifolin reduced migration in B16F10 and A375 melanoma cells by inhibiting the JNK pathway [58]. Overall, it may somehow offer an alternative or combination approach for treating metastatic melanoma cells.

As previously mentioned, the activity of MMPs and cathepsin family proteins plays a crucial role in tumour growth, local invasion and metastasis [59, 60]. The degradation of the ECM is closely linked to MMP expression, which is highly correlated with tumour progression across various melanoma subtypes [61]. For instance, overexpression of MMP‐1, MMP‐2 and MMP‐13 has been observed in nodular melanoma, a subtype characterized by aggressive growth and early metastasis [62]. In uveal melanoma, patients with positive expression of MMP‐2 and MMP‐9 have been shown to exhibit lower survival rates compared to the control group [63]. Furthermore, other studies have reported higher concentrations of MMP‐2 in metastatic melanoma (stage IV) compared to primary melanoma subgroups (stage I) [64, 65]. Taken together, these findings highlight the increased detection rates of MMP‐2 in melanoma cells.

On the other hand, when considering cathepsin proteases, the cathepsin family can be classified into aspartic proteases (CTSD, E), serine proteases (CTSA, G) and cysteine proteases (CTSB, C, F, H, K, L, O, S, V, X and W) [66]. Among these lysosomal proteases, CTSA has been shown to correlate with tumour development. For example, in lung adenocarcinoma A549 cells, knockdown of CTSA decreased invasion and migration, thereby influencing cell cycle regulators [67]. In prostate cancer, elevated levels of CTSA expression have been observed in comparison to normal prostate tissues, and suppression of CTSA genes leads to inhibition of tumour growth [68]. Similarly, previous studies have demonstrated that malignant melanoma lesions exhibit higher expression of lysosomal CTSA compared to normal pigmented nevi [69, 70]. Other studies have also suggested that UV overexposure leads to the release of cathepsins from melanoma cells into the surrounding environment [71]. In the present study, jaceidin significantly attenuates the expressions of both MMP‐2 and CTSA, both of which serve as prognostic biomarkers for predicting patient outcomes.

## 5 Conclusion

The present study revealed that jaceidin significantly reduces the motility, migration and invasion of two metastatic melanoma cell lines (HMY‐1 and A2058). It has been found to be activated by phosphorylation of the ERK and JNK pathways. Additionally, jaceidin reduces the expression of ECM degradation proteins MMP2 and CTSA. Taken together, this study identifies jaceidin as a potential anti‐migratory agent that could be used clinically to improve metastatic melanoma prognosis.

## Author Contributions

**Ming‐Ju Hsieh:** conceptualization, writing – original draft, writing – review and editing.

## Funding

The authors have nothing to report.

## Conflicts of Interest

The authors declare no conflicts of interest.

## Data Availability

The data that support the findings of this study are available from the corresponding author upon reasonable request.

## References

[1] R. L. Siegel , A. N. Giaquinto , and A. Jemal , “Cancer statistics, 2024,” Cancer Journal Clinicians 74, no. 1 (2024): 12–49, 10.3322/caac.21820. 38230766

[2] G. De Pinto , S. Mignozzi , C. La Vecchia , F. Levi , E. Negri , and C. Santucci , “Global Trends in Cutaneous Malignant Melanoma Incidence and Mortality,” Melanoma Research 34 (2024): 265, 10.1097/CMR.0000000000000959. 38391175 PMC11045545

[3] M. Rastrelli , S. Tropea , C. R. Rossi , and M. Alaibac , “Melanoma: Epidemiology, Risk Factors, Pathogenesis, Diagnosis and Classification,” In Vivo 28, no. 6 (2014): 1005–1011. 25398793

[4] Y. Qian , P. Johannet , A. Sawyers , J. Yu , I. Osman , and J. Zhong , “The Ongoing Racial Disparities in melanoma: An Analysis of the Surveillance, Epidemiology, and End Results Database (1975–2016),” Journal of the American Academy of Dermatology 84, no. 6 (2021): 1585–1593, 10.1016/j.jaad.2020.08.097. 32861710 PMC8049091

[5] C. Stefanaki , L. Chardalias , E. Soura , A. Katsarou , and A. Stratigos , “Paediatric Melanoma,” Journal of the European Academy of Dermatology and Venereology 31, no. 10 (2017): 1604–1615, 10.1111/jdv.14299. 28449284

[6] C. Garbe , T. Amaral , K. Peris , et al., “European Consensus‐Based Interdisciplinary Guideline for Melanoma. Part 1: Diagnostics: Update 2022,” European Journal of Cancer 170 (2022): 236, 10.1016/j.ejca.2022.03.008. 35570085

[7] S. E. Orme and M. D. Moncrieff , “A Review of Contemporary Guidelines and Evidence for Wide Local Excision in Primary Cutaneous Melanoma Management,” Cancers (Basel) 16 (2024): 895, 10.3390/cancers16050895. 38473257 PMC10930506

[8] J. E. Gershenwald , R. A. Scolyer , K. R. Hess , et al., “Melanoma staging: Evidence‐Based Changes in the American Joint Committee on Cancer Eighth Edition Cancer Staging Manual,” CA: a Cancer Journal for Clinicians 67, no. 6 (2017): 472–492, 10.3322/caac.21409. 29028110 PMC5978683

[9] C. Garbe , T. K. Eigentler , U. Keilholz , A. Hauschild , and J. M. Kirkwood , “Systematic Review of Medical Treatment In Melanoma: Current Status and Future Prospects,” Oncologist 16, no. 1 (2011): 5–24, 10.1634/theoncologist.2010-0190. 21212434 PMC3228046

[10] D. Pulte , J. Weberpals , L. Jansen , and H. Brenner , “Changes in Population‐Level Survival for Advanced Solid Malignancies With New Treatment Options in the Second Decade of the 21st Century,” Cancer 125, no. 15 (2019): 2656–2665, 10.1002/cncr.32160. 31095726

[11] A. Ribas , F. S. Hodi , M. Callahan , C. Konto , and J. Wolchok , “Hepatotoxicity With Combination of Vemurafenib and Ipilimumab,” New England Journal Medicine 368, no. 14 (2013): 1365–1366, 10.1056/NEJMc1302338. 23550685

[12] T. G. Soldatos , A. Dimitrakopoulou‐Strauss , L. Larribere , J. C. Hassel , and C. Sachpekidis , “Retrospective Side Effect Profiling of the Metastatic Melanoma Combination Therapy Ipilimumab‐Nivolumab Using Adverse Event Data,” Diagnostics (Basel) 8, no. 4 (2018): 76–88, 10.3390/diagnostics8040076. 30384507 PMC6316083

[13] C. Couchoud , P. Fagnoni , F. Aubin , et al., “Economic Evaluations of Cancer Immunotherapy: A Systematic Review and Quality Evaluation,” Cancer Immunology, Immunotherapy 69, no. 10 (2020): 1947–1958, 10.1007/s00262-020-02646-0. 32676716 PMC11027639

[14] Y. Tang , S. Durand , S. Dalle , and J. Caramel , “EMT‐Inducing Transcription Factors, Drivers of Melanoma Phenotype Switching, and Resistance to Treatment,” Cancers (Basel) 12, no. 8 (2020): 1031–1047, 10.3390/cancers12082154. 32759677 PMC7465730

[15] K. Wu , Y. Wei , Y. Yu , M. Shan , Y. Tang , and Y. Sun , “Green tea polyphenols inhibit malignant melanoma progression via regulating circ_MITF/miR‐30e‐3p/HDAC2 axis,” Biotechnology and Applied Biochemistry 69, no. 2 (2022): 808–821, 10.1002/bab.2153. 33797132

[16] J. Choudhari , R. Nimma , S. K. Nimal , S. K. Totakura Venkata , G. C. Kundu , and R. N. Gacche , “Prosopis Juliflora (Sw.) DC Phytochemicals Induce Apoptosis and Inhibit Cell Proliferation Signalling Pathways, EMT, Migration, Invasion, Angiogenesis and Stem Cell Markers in Melanoma Cell Lines,” Journal Ethnopharmacol 312: 116472, 10.1016/j.jep.2023.116472. 37062530

[17] R. Ronca , E. Di Salle , A. Giacomini , et al., “Long Pentraxin‐3 Inhibits Epithelial‐Mesenchymal Transition in Melanoma Cells,” Molecular Cancer Therapeutics 12, no. 12 (2013): 2760–2771, 10.1158/1535-7163.MCT-13-0487. 24130051

[18] P. Xu , M. Marsafari , J. Zha , and M. Koffas , “Microbial Coculture for Flavonoid Synthesis,” Trends in Biotechnology 38, no. 7 (2020): 686–688, 10.1016/j.tibtech.2020.01.008. 32497514

[19] A. N. Panche , A. D. Diwan , and S. R. Chandra , “Flavonoids: An Overview,” Journal of Nutritional Science 5 (2016): e47, 10.1017/jns.2016.41. 28620474 PMC5465813

[20] A. Hazafa , K. U. Rehman , N. Jahan , and Z. Jabeen , “The Role of Polyphenol (Flavonoids) Compounds in the Treatment of Cancer Cells,” Nutrition and Cancer 72, no. 3 (2020): 386–397, 10.1080/01635581.2019.1637006. 31287738

[21] J. Liu , S. M. Li , Y. J. Tang , et al., “Jaceosidin Induces Apoptosis and Inhibits Migration in AGS Gastric Cancer Cells by Regulating ROS‐Mediated Signalling Pathways,” Redox Report 29, no. 1 (2024): 2313366, 10.1080/13510002.2024.2313366. 38318818 PMC10854459

[22] Y. J. Chen , Y. J. Cheng , A. C. Hung , et al., “The Synthetic Flavonoid WYC02‐9 Inhibits Cervical Cancer Cell Migration/Invasion and Angiogenesis via MAPK14 Signalling,” Gynecologic Oncology 131, no. 3 (2013): 734–743, 10.1016/j.ygyno.2013.10.012. 24145114

[23] S. S. Elhady , E. E. Eltamany , A. E. Shaaban , et al., “Jaceidin Flavonoid Isolated from Chiliadenus Montanus Attenuates Tumour Progression in Mice via VEGF Inhibition: In Vivo and In Silico Studies,” Plants (Basel) 9, no. 8 (2020), 10.3390/plants9081031. PMC7464537 32823927

[24] Z. Gharari , P. Hanachi , and T. R. Walker , “Green Synthesised Ag‐Nanoparticles Using Scutellaria Multicaulis Stem Extract and Their Selective Cytotoxicity Against Breast Cancer,” Anal Biochem 653, (2022): 114786, 10.1016/j.ab.2022.114786. 35714944

[25] G. J. Todaro , C. Fryling , and J. E. De Larco , “Transforming Growth Factors Produced by Certain Human Tumour Cells: Polypeptides That Interact With Epidermal Growth Factor Receptors,” Proceedings of the National Academy of Sciences of the United States of America 77, no. 9 (1980): 5258–5262, 10.1073/pnas.77.9.5258. 6254071 PMC350037

[26] S. Kondo , N. Sato , K. Sato , Y. Hozumi , and K. Aso , “Establishment of Cell Lines From Human Malignant Melanomas and Their Application to the Assessment of Natural Killer‐Like Cytotoxicity,” Journal of Dermatology 11, no. 4 (1984): 328–334, 10.1111/j.1346-8138.1984.tb01486.x. 6239887

[27] Y. Sun , W. Z. Liu , T. Liu , X. Feng , N. Yang , and H. F. Zhou , “Signalling Pathway of MAPK/ERK in Cell Proliferation, Differentiation, Migration, Senescence and Apoptosis,” Journal of Receptor and Signal Transduction Research 35, no. 6 (2015): 600–604, 10.3109/10799893.2015.1030412. 26096166

[28] V. Mittal , “Epithelial Mesenchymal Transition in Tumour Metastasis,” Annual Review of Pathology 13 (Jan 24 2018): 395–412, 10.1146/annurev-pathol-020117-043854. 29414248

[29] I. Pastushenko and C. Blanpain , “EMT Transition States During Tumour Progression and Metastasis,” Trends in Cell Biology 29, no. 3 (2019): 212–226, 10.1016/j.tcb.2018.12.001. 30594349

[30] S. Usman , N. H. Waseem , T. K. N. Nguyen , et al., “Vimentin is at the Heart of Epithelial Mesenchymal Transition (EMT) Mediated Metastasis,” Cancers (Basel) 13 (2021): 85, 10.3390/cancers13194985. PMC8507690 34638469

[31] S. Brabletz , H. Schuhwerk , T. Brabletz , and EMT , “Dynamic: A Multi‐Tool for Tumour Progression,” EMBO J 40 (2021): 108647, 10.15252/embj.2021108647. PMC8441439 34459003

[32] Y. Wang , J. Shi , K. Chai , X. Ying , and B. P. Zhou , “The Role of Snail in EMT and Tumorigenesis,” Current Cancer Drug Targets 13, no. 9 (2013): 963–972, 10.2174/15680096113136660102. 24168186 PMC4004763

[33] R. Fontana and J. Yang , “Matrix Degradation Assay to Measure the Ability of Tumour Cells to Degrade Extracellular Matrix,” Methods in Molecular Biology 2294 (2021): 151–163, 10.1007/978-1-0716-1350-4_11. 33742400

[34] C. Gialeli , A. D. Theocharis , and N. K. Karamanos , “Roles of Matrix Metalloproteinases in Cancer Progression and Their Pharmacological Targeting,” FEBS Journal 278, no. 1 (2011): 16–27, 10.1111/j.1742-4658.2010.07919.x. 21087457

[35] H. H. Lin , S. J. Chen , M. R. Shen , et al., “Lysosomal Cysteine Protease Cathepsin S is Involved in Cancer Cell Motility by Regulating Store‐Operated Ca(2+) Entry,” Biochimica et Biophysica Acta (BBA) ‐ Molecular Cell Research 1866, no. 12 (2019): 118517, 10.1016/j.bbamcr.2019.07.012. 31340164

[36] H. Jiang and H. Li , “Prognostic Values Of Tumoral Mmp2 And Mmp9 Overexpression In Breast Cancer: A Systematic Review And Meta‐Analysis,” BMC Cancer 21 (2021): 149, 10.1186/s12885-021-07860-2. 33568081 PMC7877076

[37] R. Li , X. Song , Y. Guo , P. Song , D. Duan , and Z. S. Chen , “Natural Products: A Promising Therapeutics for Targeting Tumour Angiogenesis,” Frontiers in Oncology 11 (2021): 772915, 10.3389/fonc.2021.772915. 34746014 PMC8570131

[38] E. R. Sauter , “Cancer Prevention and Treatment Using Combination Therapy With Natural Compounds,” Expert Review of Clinical Pharmacology 13, no. 3 (2020): 265–285, 10.1080/17512433.2020.1738218. 32154753

[39] A. Parveen , R. Sultana , S. M. Lee , T. H. Kim , and S. Y. Kim , “Phytochemicals Against Anti‐Diabetic Complications: Targeting The Advanced Glycation end Product Signalling Pathway,” Archives of Pharmacal Research 44, no. 4 (2021): 378–401, 10.1007/s12272-021-01323-9. 33837513

[40] J. Wu , T. Zhou , Y. Wang , Y. Jiang , and Y. Wang , “Mechanisms and Advances in Anti‐Ovarian Cancer with Natural Plants Component,” Molecules 26, no. 4 (2024): 308, 10.3390/molecules26195949. PMC8512305 34641493

[41] R. Kalluri and R. A. Weinberg , “The Basics of Epithelial‐Mesenchymal Transition,” Journal of Clinical Investigation 119, no. 6 (2009): 1420–1428, 10.1172/JCI39104. 19487818 PMC2689101

[42] S. M. Ridge , F. J. Sullivan , and S. A. Glynn , “Mesenchymal Stem Cells: Key Players In Cancer Progression,” Molecular Cancer 16 (2017): 31, 10.1186/s12943-017-0597-8. 28148268 PMC5286812

[43] D. Pedri , P. Karras , E. Landeloos , J. C. Marine , and F. Rambow , “Epithelial‐To‐Mesenchymal‐Like Transition Events in Melanoma,” FEBS Journal 289, no. 5 (2022): 1352–1368, 10.1111/febs.16021. 33999497

[44] D. Medici , E. D. Hay , and B. R. Olsen , “Snail and Slug Promote Epithelial‐Mesenchymal Transition Through Beta‐Catenin‐T‐cell Factor‐4‐Dependent Expression of Transforming Growth Factor‐Beta3,” Molecular Biology of the Cell 19, no. 11 (2008): 4875–4887, 10.1091/mbc.e08-05-0506. 18799618 PMC2575183

[45] N. K. Kurrey , S. P. Jalgaonkar , A. V. Joglekar , et al., “Snail and Slug Mediate Radioresistance And Chemoresistance By Antagonising P53‐Mediated Apoptosis and Acquiring A Stem‐Like Phenotype In Ovarian Cancer Cells,” Stem Cells 27, no. 9 (2009): 2059–2068, 10.1002/stem.154. 19544473

[46] Q. Guo , F. Ning , R. Fang , et al., “Endogenous Nodal Promotes Melanoma Undergoing Epithelial‐Mesenchymal Transition via Snail and Slug In Vitro and In Vivo,” American Journal of Cancer Research 5, no. 6 (2015): 2098–2112. 26269769 PMC4529629

[47] J. Y. Fang and B. C. Richardson , “The MAPK Signalling Pathways and Colorectal Cancer,” Lancet Oncology 6, no. 5 (2005): 322–327, 10.1016/S1470-2045(05)70168-6. 15863380

[48] E. K. Kim and E. J. Choi , “Pathological Roles of MAPK Signalling Pathways in Human Diseases,” Biochimica et Biophysica Acta 1802, no. 4 (2010): 396–405, 10.1016/j.bbadis.2009.12.009. 20079433

[49] J. Dorrie , L. Babalija , S. Hoyer , et al., “BRAF and MEK Inhibitors Influence the Function of Reprogrammed T Cells: Consequences for Adoptive T‐Cell Therapy,” International Journal of Molecular Sciences 19, no. 1 (2018): 289–304, 10.3390/ijms19010289. 29346301 PMC5796234

[50] J. N. Sanchez , T. Wang , and M. S. Cohen , “BRAF and MEK Inhibitors: Use and Resistance in BRAF‐Mutated Cancers,” Drugs 78, no. 5 (2018): 549–566, 10.1007/s40265-018-0884-8. 29488071 PMC6080616

[51] P. Savoia , P. Fava , F. Casoni , and O. Cremona , “Targeting the ERK Signalling Pathway in Melanoma,” International Journal of Molecular Sciences 20 (2019): 483, 10.3390/ijms20061483. 30934534 PMC6472057

[52] P. Savoia , E. Zavattaro , and O. Cremona , “Clinical Implications of Acquired BRAF Inhibitors Resistance in Melanoma,” International Journal of Molecular Sciences 21, no. 24 (2020): 9730–9744, 10.3390/ijms21249730. 33419275 PMC7766699

[53] S. J. Welsh , H. Rizos , R. A. Scolyer , and G. V. Long , “Resistance to Combination BRAF and MEK Inhibition in Metastatic Melanoma: Where to Next?,” European Journal of Cancer 62 (2016): 76–85, 10.1016/j.ejca.2016.04.005. 27232329

[54] J. Xiao , M. E. Egger , K. M. McMasters , and H. Hao , “Differential Expression Of Abcb5 In Braf Inhibitor‐Resistant Melanoma Cell Lines,” BMC Cancer 18 (2018): 675, 10.1186/s12885-018-4583-3. 29929490 PMC6014033

[55] K. Zhao , Q. Dai , J. Wu , Z. Wei , Y. Duan , and B. Chen , “Morusin Enhances the Antitumor Activity of MAPK Pathway Inhibitors in BRAF‐Mutant Melanoma by Inhibiting the Feedback Activation of STAT3,” European Journal of Cancer 165 (2022): 58–70, 10.1016/j.ejca.2022.01.004. 35219024

[56] L. Zhang , S. Meng , B. Yan , et al., “Anti‐Proliferative, Pro‐Apoptotic, Anti‐Migrative and Tumour‐Inhibitory Effects and Pleiotropic Mechanism of Theaflavin on B16F10 Melanoma Cells,” Oncotargets and Therapy 14 (2021): 1291–1304, 10.2147/OTT.S286350. 33658796 PMC7920628

[57] Y. H. Han , J. G. Mun , H. D. Jeon , J. Park , J. Y. Kee , and S. H. Hong , “Gomisin A Ameliorates Metastatic Melanoma By Inhibiting Ampk And Erk/Jnk‐Mediated Cell Survival and Metastatic Phenotypes,” Phytomedicine 68 (2020): 153147, 10.1016/j.phymed.2019.153147. 32028184

[58] L. Xu , L. Zhang , S. Zhang , et al., “Taxifolin Inhibits Melanoma Proliferation/Migration Impeding Usp18/Rac1/Jnk/Beta‐Catenin Oncogenic Signalling,” Phytomedicine 123 (2024): 155199, 10.1016/j.phymed.2023.155199. 37995531

[59] S. Niland , A. X. E. Riscanevo , and J. A. Eble , “Matrix Metalloproteinases Shape the Tumour Microenvironment in Cancer Progression,” International Journal of Molecular Sciences 23 (2021): 146, 10.3390/ijms23010146. 35008569 PMC8745566

[60] V. Gocheva , H. W. Wang , B. B. Gadea , et al., “Il‐4 Induces Cathepsin Protease Activity In Tumour‐Associated Macrophages To Promote Cancer Growth And Invasion,” Genes Development 24 (2010): 241, 10.1101/gad.1874010. 20080943 PMC2811826

[61] Y. R. Liu , B. Sun , X. L. Zhao , et al., “Basal Caspase‐3 Activity Promotes Migration, Invasion, and Vasculogenic Mimicry Formation of Melanoma Cells,” Melanoma Research 23, no. 4 (2013): 243–253, 10.1097/CMR.0b013e3283625498. 23695439

[62] G. Zamolo , M. Grahovac , G. Zauhar , et al., “Matrix Metalloproteinases MMP‐1, MMP‐2, and MMP‐13 are Overexpressed in Primary Nodular Melanoma,” Journal of Cutaneous Pathology 47, no. 2 (2020): 139–145, 10.1111/cup.13603. 31677173

[63] Y. El‐Shabrawi , N. Ardjomand , H. Radner , and N. Ardjomand , “MMP‐9 is Predominantly Expressed in Epithelioid and Not Spindle Cell Uveal Melanoma,” Journal of Pathology 194, no. 2 (2001): 201, 10.1002/1096-9896. 11400149

[64] P. Redondo , P. Lloret , M. Idoate , and S. Inoges , “Expression and Serum Levels of MMP‐2 and MMP‐9 During Human Melanoma Progression,” Clinical and Experimental Dermatology 30, no. 5 (2005): 541–545, 10.1111/j.1365-2230.2005.01849.x. 16045689

[65] Y. Ohnishi , S. Tajima , and A. Ishibashi , “Coordinate Expression of Membrane Type‐Matrix Metalloproteinases‐2 and 3 (MT2‐MMP and MT3‐MMP) and Matrix Metalloproteinase‐2 (MMP‐2) In Primary And Metastatic Melanoma Cells,” European Journal of Dermatology 11, no. 5 (2001): 420–423. 11525948

[66] V. Turk , V. Stoka , O. Vasiljeva , et al., “Cysteine Cathepsins: From Structure, Function And Regulation To New Frontiers,” Journal of Biochemistry and Biophysics 1824, no. 1 (2012): 68–88, 10.1016/j.bbapap.2011.10.002. PMC7105208 22024571

[67] B. Hu , X. Zhu , and J. Lu , “Cathepsin A Knockdown Decreases the Proliferation and Invasion of A549 Lung Adenocarcinoma Cells,” Molecular Medicine Reports 21, no. 6 (2020): 2553–2559, 10.3892/mmr.2020.11068. 32323791 PMC7185279

[68] S. Park , W. Kwon , J. K. Park , et al., “Suppression of Cathepsin a Inhibits Growth, Migration, and Invasion by Inhibiting the p38 MAPK Signalling Pathway in Prostate Cancer,” Archives of Biochemistry and Biophysics 688 (2020): 108407, 10.1016/j.abb.2020.108407. 32407712

[69] L. Kozlowski , M. Z. Wojtukiewicz , and H. Ostrowska , “Cathepsin A Activity in Primary And Metastatic Human Melanocytic Tumours,” Archives of Dermatological Research 292, no. 2–3 (2000): 68–71, 10.1007/s004030050012. 10749558

[70] E. Frohlich , “Proteases in Cutaneous Malignant Melanoma: Relevance As Biomarker and Therapeutic Target,” Cellular and Molecular Life Sciences 67, no. 23 (2010): 3947–3960, 10.1007/s00018-010-0469-5. 20686912 PMC11115755

[71] C. B. Eding , J. Domert , P. Waster , F. Jerhammar , I. Rosdahl , and K. Ollinger , “Melanoma Growth and Progression After Ultraviolet a Irradiation: Impact of Lysosomal Exocytosis and Cathepsin Proteases,” Acta Dermato‐Venereologica 95, no. 7 (2015): 792–797, 10.2340/00015555-2064. 25669167

